# *RGS2* expression predicts amyloid-β sensitivity, MCI and Alzheimer's disease: genome-wide transcriptomic profiling and bioinformatics data mining

**DOI:** 10.1038/tp.2016.179

**Published:** 2016-10-04

**Authors:** A Hadar, E Milanesi, A Squassina, P Niola, C Chillotti, M Pasmanik-Chor, O Yaron, P Martásek, M Rehavi, D Weissglas-Volkov, N Shomron, I Gozes, D Gurwitz

**Affiliations:** 1 Department of Human Molecular Genetics and Biochemistry, Sackler Faculty of Medicine, Tel Aviv University, Tel Aviv, Israel; 2 Department of Biomedical Sciences, University of Cagliari, Cagliari, Italy; 3 Unit of Clinical Pharmacology, University Hospital of Cagliari, Cagliari, Italy; 4 Bioinformatics Unit, George Wise Faculty of Life Sciences, Tel Aviv University, Tel Aviv, Israel; 5 The Genomic Analysis Laboratory, Sackler Faculty of Medicine, Tel Aviv University, Tel Aviv, Israel; 6 Department of Pediatrics and Adolescent Medicine, First Faculty of Medicine, Charles University in Prague and General University Hospital in Prague, Prague, Czech Republic; 7 Department of Physiology and Pharmacology, Sackler Faculty of Medicine, Tel Aviv University, Ramat Aviv, Tel Aviv, Israel; 8 Department of Cell and Developmental Biology, Sackler Faculty of Medicine, Tel Aviv University, Tel Aviv, Israel; 9 Adams Super Center for Brain Studies, Sagol School of Neuroscience, Tel Aviv University, Tel Aviv, Israel

## Abstract

Alzheimer's disease (AD) is the most frequent cause of dementia. Misfolded protein pathological hallmarks of AD are brain deposits of amyloid-β (Aβ) plaques and phosphorylated tau neurofibrillary tangles. However, doubts about the role of Aβ in AD pathology have been raised as Aβ is a common component of extracellular brain deposits found, also by *in vivo* imaging, in non-demented aged individuals. It has been suggested that some individuals are more prone to Aβ neurotoxicity and hence more likely to develop AD when aging brains start accumulating Aβ plaques. Here, we applied genome-wide transcriptomic profiling of lymphoblastoid cells lines (LCLs) from healthy individuals and AD patients for identifying genes that predict sensitivity to Aβ. Real-time PCR validation identified 3.78-fold lower expression of *RGS2* (regulator of G-protein signaling 2; *P*=0.0085) in LCLs from healthy individuals exhibiting high vs low Aβ sensitivity. Furthermore, *RGS2* showed 3.3-fold lower expression (*P*=0.0008) in AD LCLs compared with controls. Notably, *RGS2* expression in AD LCLs correlated with the patients' cognitive function. Lower *RGS2* expression levels were also discovered in published expression data sets from postmortem AD brain tissues as well as in mild cognitive impairment and AD blood samples compared with controls. In conclusion, Aβ sensitivity phenotyping followed by transcriptomic profiling and published patient data mining identified reduced peripheral and brain expression levels of *RGS2*, a key regulator of G-protein-coupled receptor signaling and neuronal plasticity. *RGS2* is suggested as a novel AD biomarker (alongside other genes) toward early AD detection and future disease modifying therapeutics.

## Introduction

Alzheimer's disease (AD), a progressive neurodegenerative disorder, is the most frequent cause of dementia. Old age is a major AD risk factor: the annual AD incidence increases from 1% between ages of 60 and 70 years to 6–8% at the age of 85 or older.^1, 2^ AD is characterized by misfolded protein pathological brain hallmarks: extracellular deposits of amyloid-β (Aβ) plaques and accumulation of phosphorylated tau neurofibrillary tangles. The Aβ_1__–42_ peptide aggregates are predominant in AD brain plaques and considered the most neurotoxic Aβ form.^3, 4, 5, 6, 7^ However, there are individuals who exhibit Aβ plaques in the absence of dementia symptoms.^1, 8, 9, 10^ Mild cognitive impairment (MCI) is a state when there is mild loss of memory, considered normal for old age. Fifty percent of MCI patients will progress to AD over 4 years.^1^

Efforts have been made for identifying early AD biomarkers that may detect high-risk individuals so that they are prioritized for disease-modifying drugs that are being developed.^11, 12^ Imaging techniques based on *in vivo* measurements of brain Aβ have been disappointing,^13^ and indeed one of the biggest mysteries in AD pathophysiology is that some aged individuals show, upon brain imaging, large quantities of brain Aβ deposits without showing clinical AD signs and while maintaining good cognitive skills into their 80s.^13^ This has recently led to strong doubts about the validity of the ‘amyloid cascade hypothesis' that assumes a central role for Aβ in AD pathology.^14, 15^ It has been proposed that some individuals could be more prone to Aβ-mediated neurotoxicity, while Aβ brain deposition *per se* may represent part of the normal brain aging process.^13, 16^

To further understand the pathophysiology of AD toward potential prevention and disease-modifying treatments, disease biomarkers may prove beneficial. One approach is the candidate gene approach, which we (IG) recently took, finding correlation between serum activity-dependent neuroprotective protein (ADNP) and intelligence test scores of elderly individuals, coupled with lower ADNP messenger RNA (mRNA) in blood cells correlated with increased Aβ deposits and significant deregulation of activity-dependent neuroprotective protein mRNA expression in AD lymphocytes.^17^ Another approach entails proteomic screening.^18, 19^ In our present work, we applied a third approach, namely, genome-wide transcriptomics of human lymphoblastoid cell lines (LCLs) from unrelated healthy individuals and AD patients for searching gene expression levels that are correlated with *in vitro* Aβ sensitivity. We report several genes, most notably *RGS2* (regulator of G-protein signaling 2) and *DLGAP1* (disks, large (Drosophila) homolog-associated protein 1) with low expression correlated with higher Aβ sensitivity in LCLs from healthy individuals and lower expression in LCLs from AD patients, as well as in postmortem AD brain tissues and both AD and MCI peripheral blood.

## Materials and methods

### Human LCLs and materials

LCLs from adult donors were obtained from the National Laboratory for the Genetics of Israeli Populations (NLGIP; http://nlgip.tau.ac.il) at Tel Aviv University, Israel (23 LCLs of healthy controls) and from The University of Cagliari, Italy (28 AD patients and 16 healthy controls). Detailed demographic data and cognitive scores of the AD patients and controls are presented in Supplementary Table 1. The cell lines were generated from peripheral blood lymphocytes donated by consenting patients and healthy controls. The cells were maintained in optimal growth conditions as described.^20^ Tissue-culture reagents were purchased from Biological Industries (Beit-Haemek, Israel). Amyloid-β_1__–42_ (Aβ_1__–__42_) peptide was purchased from Genemed Synthesis (San Antonio, TX, USA). Aβ_1__–42_ peptide was dissolved in sterile tissue-culture grade water (1 mg ml^−1^) and stored (as 100 μl aliquots) at −20 °C. Before the experiments, an aliquot of Aβ_1__–42_ in water was preincubated at 37 °C for 3 days^21, 22^ for assuring the generation of Aβ fibrils.^23, 24^

### Cell proliferation assay

Growth inhibition of LCLs was examined by exposure to 8 μm Aβ_1__–42_ fibrils for 3 days (unless otherwise indicated). LCLs were first washed in phosphate-buffered saline and suspended with serum-free RPMI medium containing the commercial serum supplement 4% BIOGRO-2 (Biological Industries). This BIOGRO-2 concentration was optimal for long-term serum-free growth of LCLs.^25^ The serum-free conditions are essential for observing Aβ_1__–42_ mediated growth inhibition. The cells were counted and diluted in the same media to a concentration of 250 000 cells ml^−1^, followed by plating 100 μl per well in 96-well plates (Corning, Corning, NY, USA). The LCLs from healthy controls or AD patients were similarly assayed. The XTT cell proliferation assay (Biological Industries) was carried out after 3 days, as earlier described.^26^ Each cell line was tested for the effect of Aβ_1__–42_ in three independent experiments.

### RNA extraction

RNA extraction was performed from cells incubated in upright T-25 flasks under optimal growth conditions in serum-containing media at a cell density of 0.5 × 10^6^–1 × 10^6^ cells ml^−1^ as previously described.^20^ RNA was quantified using a NanoDrop spectrophotometer (NanoDrop, Wilmington, DE, USA), with both 260/280 nm and 260/230 nm parameters >2.0. RNA quality was confirmed using 1% agarose gels.

### Gene expression microarrays

The RNA samples (*N*=16) from optimally growing LCLs, exhibiting high or low sensitivities to Aβ (8 each) were chosen for genome-wide expression profiling. The RNA samples (250 ng) were prepared and hybridized to Affymetrix Human Gene 2.1 ST arrays as described in the Affymetrix website. Microarray analysis was performed on CEL files using Partek Genomics Suite (Partek, Chesterfield, MO, USA). Genes of interest that were differentially expressed in the two phenotypic groups of the LCLs (fold-difference cutoff >1.5 and *P*<0.05) were obtained.

### Real-time PCR

Real-time PCR was performed to validate the microarray expression patterns of selected genes using the same RNA samples used for the microarray experiment. The complementary DNA (cDNA) samples were prepared from 1 μg RNA samples using High Capacity cDNA Reverse Transcription kit (Applied Biosystems, Waltham, MA, USA) containing 10 × RT buffer, 10 × RT random primers, 25 × dNTP mix, RNAse inhibitor and MultiScribe Reverse transcriptase. Reverse transcription was performed using a thermal cycler over three steps (25 °C for 10 min, followed by 37 °C for 120 min and 85 °C for 5 min). Real-time PCR experiments were done with 20 μl mixtures containing 20 ng of cDNA, Absolute Blue qPCR ROX mix (Thermo Scientific, Waltham, MA, USA) and Primers (TaqMan Gene Expression Assay; Applied Biosystems). *GUSB* (glucuronidase, beta) was used as reference gene as recommended for transcriptomic analysis of LCLs.^27^ TaqMan Gene Expression Assay IDs are listed below:


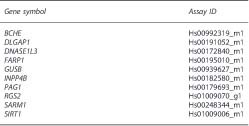


PCR reactions were performed using ABI Step One (Applied Biosystems) and the cycle protocol was as follows: 50 °C for 2 min, 95 °C for 15 min, followed by 40 cycles of 95 °C for 15 s and 60 °C for 1 min. Comparative critical threshold (Ct) values were used for analyzing relative gene expression in selected sample groups according to 2^−ΔCт^ (ΔCт=Ct target Gene—Ct reference gene). For SNORD116-13 and for the reference gene *GUSB*, real-time PCR was done using the SYBR Green (Kapa SYBR, Wilmington, MA, USA) technique. Primers (shown below) were purchased from IDT (Coralville, IA, USA).


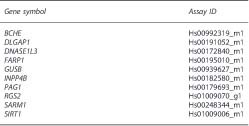


### GEO data mining

The NCBI Gene Expression Omnibus (GEO) was searched for expression data sets of human AD and MCI blood and postmortem brain tissues. Data sets GSE5281 (ref. 28) from six postmortem brain regions (87 AD and 74 controls), and GSE63060 (ref. 29) from whole blood (145 AD, 80 MCI and 104 controls) were identified as the largest cohorts. GEO files were downloaded using the R package GEO. The five selected candidate genes were tested for differential expression between AD, MCI and controls using the R package Limma.

## Results

**Figure 1:**
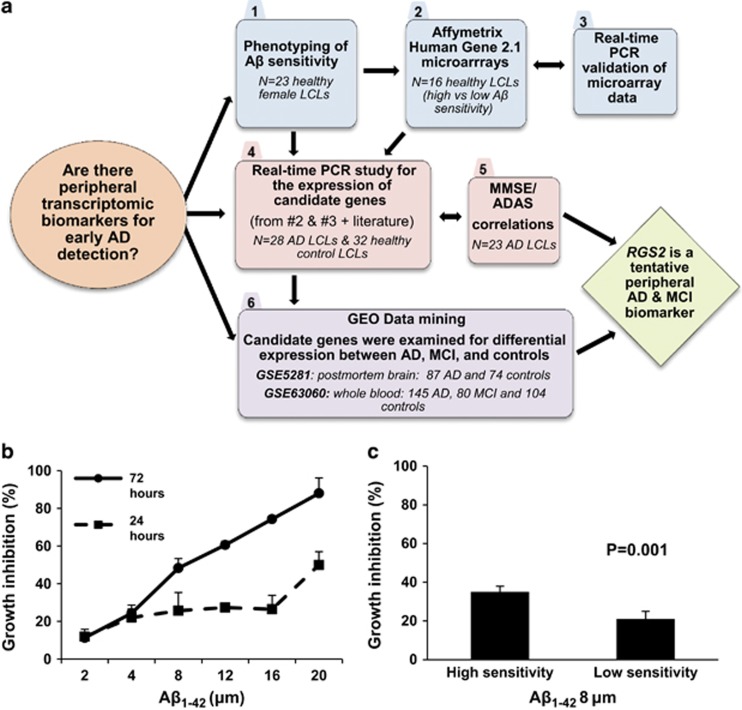
(**a**) A flowchart presenting the study design. Aβ_1__–42_ sensitivity determined in lymphoblastoid cells lines (LCLs) of healthy female donors. (**b**) Lymphoblastoid cells from a healthy female donor were plated in 96-well plates (25 000 cells per well) and incubated with the indicated concentrations of Aβ_1__–42_ for 24 or 72 h followed by determination of viable cell numbers with the XTT color reagent (see the 'Materials and methods' section). Data are from a representative experiment, with similar observations obtained in a repeat experiment. (**c**) Aβ_1__–42_ sensitivity (8 μm, 72 h) is shown for two LCL groups (eight unrelated donors each) from healthy female donors selected for the microarray experiment based on their different Aβ_1__–42_ sensitivity phenotypes. Average growth inhibition values were 35±4 and 21±3 in the high- and low-sensitivity groups, respectively (*P*=0.001; Mann–Whitney *U*-test). See the 'Materials and methods' section for experimental protocol. AD, Alzheimer's disease; ADAS, Alzheimer's Disease Assessment Scale; MCI, mild cognitive impairment; MMSE, Mini Mental State Examination.

A flowchart outlining our study design is presented in Figure 1a. As a preparatory step for genome-wide transcriptomic search for genes implicated in Aβ_1__–42_ sensitivity of cells from unrelated individuals, we initially screened human LCLs from healthy female donors for growth inhibition following incubation with several concentrations of Aβ_1__–42_ (range 1 to 20 μm) for 24 or 72 h in serum-free medium (see the Materials and methods' section). This first phase included exclusively female LCLs, as sex was shown to affect gene expression by human LCLs.^30^ Confirming previous reports, Aβ_1__–42_ did not grossly affect cell growth or survival in serum-containing media^22^ (Figure 1b). Thus, the concentration of 8 μm Aβ_1__–42_ and the incubation period of 72 h in serum-free medium were selected for phenotyping Aβ-mediated growth inhibition in a panel of LCLs from 23 unrelated healthy female donors using XTT cell proliferation assay (see the 'Materials and methods' section). Three repeat experiments were performed (each in triplicate) for each cell line. Eight LCLs exhibiting the highest Aβ_1__–42_ sensitivity (35±3% growth inhibition) and eight LCLs exhibiting the lowest Aβ_1__–42_ sensitivity (21±4% growth inhibition; Figure 1c) were selected for comparative genome-wide expression profiling (see the 'Materials and methods' section; step #1, Figure 1a). Average donor ages were similar for the high and low Aβ_1__–42_ sensitivity groups (38±8 and 58±10 years, respectively; *P*=0.142).

### Genome-wide microarray expression and RT-PCR validation

The RNA samples were prepared from the 16 selected healthy female LCLs growing under optimal conditions in serum-containing medium (see the 'Materials and methods' section). Genome-wide expression profiles were compared in the healthy donor LCLs exhibiting high or low Aβ_1__–42_ sensitivity (*n*=8 per group) using Affymetrix Human Gene 2.1 ST arrays (See the 'Materials and methods' section; step #2, Figure 1a). Table 1 shows 27 transcripts found to exhibit >1.5 fold difference (*P*<0.05) in basal expression levels comparing healthy female LCLs exhibiting high vs low Aβ_1__–42_ sensitivity.

**Figure 2:**
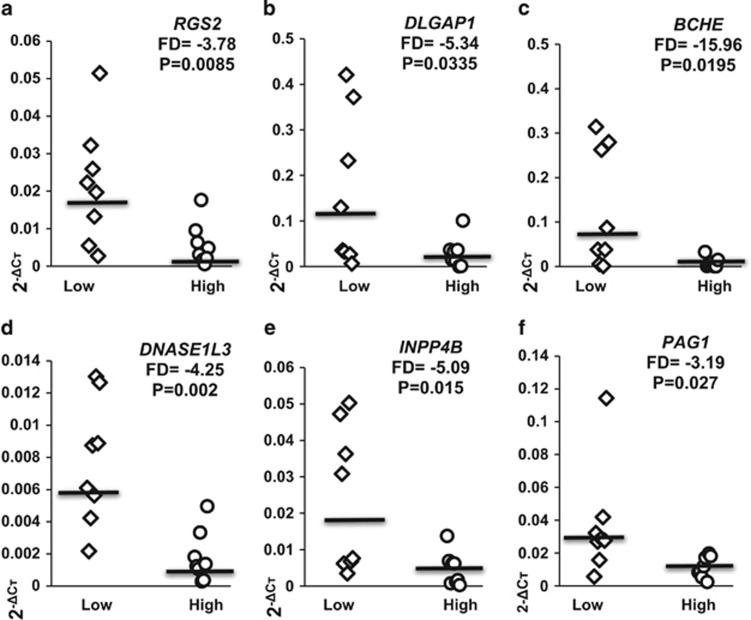
Differences in the expression levels of *RGS2, DLGAP1, BCHE*, *DNASE1L3, INPP4B,* and *PAG1* as determined by real-time PCR in two groups of healthy female lymphoblastoid cells lines (LCLs). Fold-difference (FD) values for real-time PCR validation of the microarray experiment are shown for LCLs exhibiting high vs low Aβ_1__–42_ sensitivity (eight in each group; Figure 1) FD and *P*-values are shown for (**a**) *RGS2*; (**b**) *DLGAP1*; (**c**) *BCHE*; (**d**) *DNASE1L3*; (**e**) *INPP4B and* (**f**) *PAG1.* Note: SNORD116-13 and *FARP1*, two genes from the eight selected for real-time PCR validation (from those depicted in bold font in Table 1) showed the expected trend, however, with *P*>0.05 and their expression data are not displayed.

The same RNA samples from the LCLs exhibiting high or low Aβ_1__–42_ sensitivity (eight in each group) were converted to cDNA. Eight genes from the 27 found as differentially expressed were selected for validation by real-time PCR based on their high expression in human brain tissues as well as their relevance for neuronal function. The expression levels of *BCHE*, *DLGAP1, INPP4B, DNASE1L3, RGS2 and PAG1* (Table 1) are presented as scatter plots (Figures 2a–f). The expression level differences between the two Aβ_1__–__42_ sensitivity groups of control LCLs are clearly evident.

### Expression levels of candidate genes in AD vs healthy control LCLs

**Figure 3:**
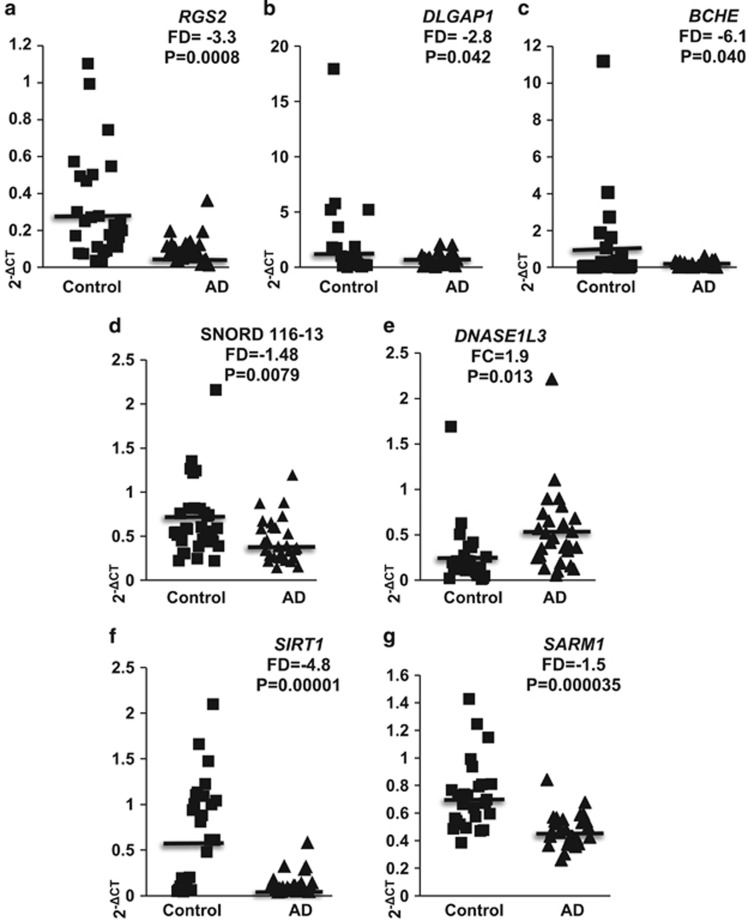
Expression levels of *RGS2, DLGAP1, BCHE,* SNORD116-13*, DNASE1L3, SIRT1 and SARM1* in lymphoblastoid cells lines (LCLs) of Alzheimer's disease (AD) patients and healthy controls. The values were determined by real-time PCR; fold-difference (FD) values are shown for 2^−ΔCT^ (see the 'Materials and methods' section). (**a**) *RSG2*: AD (*N*=28) and control (*N*=32). (**b**) *DLGAP1*: AD (*N*=24) and control (*N*=28). (**c**) *BCHE*: AD (*N*=27) and control (*N*=28). (**d**) SNORD116-13: AD (*N*=28) and control (*N*=32). (**e**) *DNASE1L3*: AD (*N*=28) and control (*N*=32). (**f**) *SIRT1*: AD (*N*=28) and control (*N*=32). (**g**) *SARM1*: AD (*N*=28) and control (*N*=32).

Next, the expression levels of selected genes found to be differentially expressed in healthy control LCLs with high vs low Aβ_1__–42_ sensitivity were determined by real-time PCR in 28 AD and 32 healthy control LCLs growing under optimal conditions (see the 'Materials and methods' section). The expression levels of two additional genes, *SIRT1* and *SARM1*, albeit not found in our genome-wide transcriptomic experiment, were also analyzed in the same AD and healthy control LCLs, as both have been implicated in AD,^31, 32, 33, 34, 35^ and *SIRT1* expression was reduced in postmortem AD parietal cortex.^36^ The RNA samples were extracted and converted to cDNA for determining the expression levels of selected genes by real-time PCR (Supplementary Table 2). The expression levels of *RGS2*, *DLGAP1*, *BCHE*, SNORD116-13, *DNASE1L3*, *SIRT1* and *SARM1* are also presented as scatter plots (Figures 3a–g). No correlations were observed between expression levels of *RGS2*, *DLGAP1* or *BCHE* and between control or AD patient ages, or between ages and growth inhibition by 8 μm Aβ_1__–42_ in individual LCLs (Supplementary Figure 1). However, a correlation was observed between the expression levels of *RGS2* and growth inhibition by Aβ_1__–42_ in 26 individual healthy control LCLs (*R*=−0.565; *P*=0.003) but not in 32 AD LCLs (Supplementary Figure 2). In addition, a correlation (*R*=0.688; *P*=0.000000006) was found between the expression levels of *DLGAP1* and *BCHE* in individual LCLs from pooled 55 control and AD LCLs (Supplementary Figure 3). Correlations were also found between the expression levels of *SIRT1* and the expression of *RGS2* or *SARM1* in individual LCLs (Supplementary Figure 4).

### *RGS2* expression levels in LCLs correlate with dementia scores

**Figure 4:**
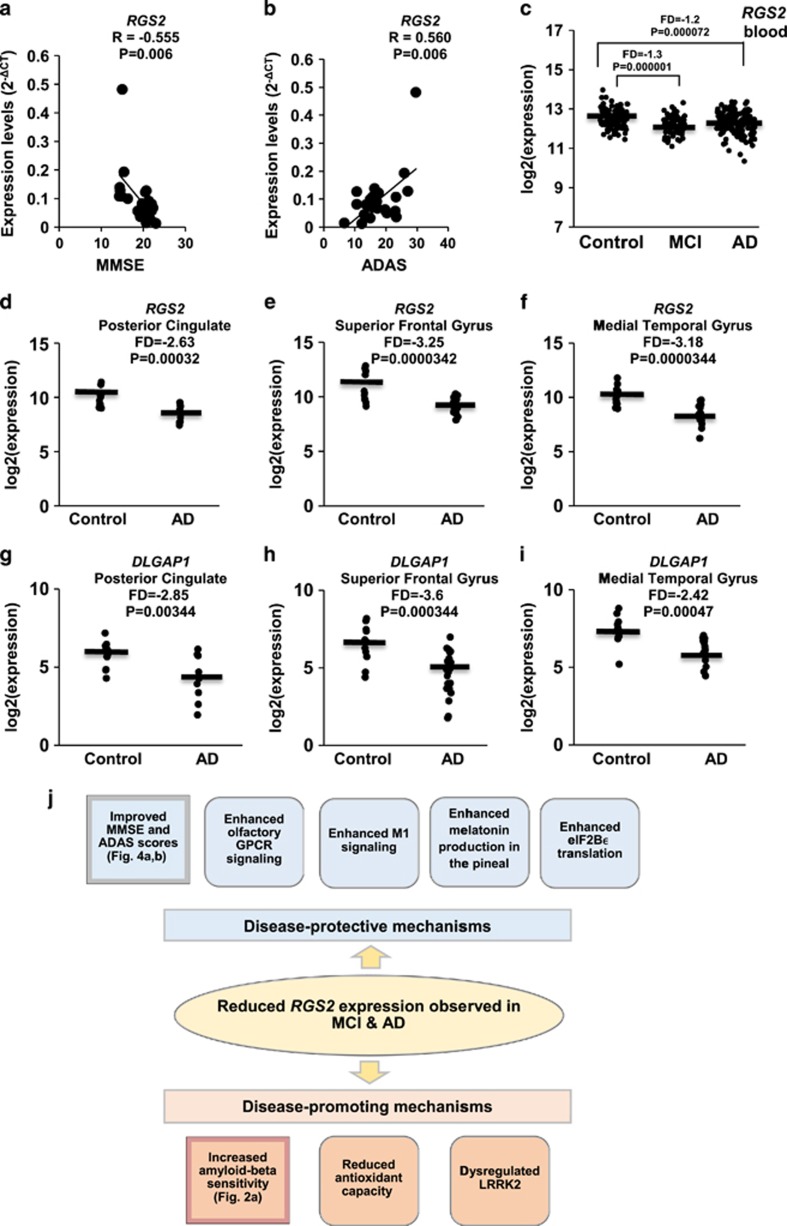
(**a** and **b**) Correlations between the expression levels of *RGS2* in lymphoblastoid cells lines (LCLs) from 23 individual Alzheimer's disease (AD) patients (Mini Mental State Examination (MMSE) <24) and their cognitive test scores as determined at the time of blood withdrawal for LCL generation. Note that higher MMSE scores reflect better cognition, whereas it is the opposite for Alzheimer's Disease Assessment Scale (ADAS) scores (**a**) MMSE: a negative correlation of *R*=−0.555 (*P*=0.006) was observed (**b**) ADAS: a positive correlation of *R*=0.560 (*P*=0.006) was observed. Note: MMSE<24 scores were determined for 23 out of 28 AD patients (Supplementary Table 2). (**c**) Expression levels of *RGS2* in 80 mild cognitive impairment (MCI), 104 healthy controls and 145 AD patients from whole blood (Data set GSE63060) (**d**–**i**) Expression levels of *RGS2* and *DLGAP1* in postmortem brains (Data set GSE5281) from AD patients and age-matched non-demented controls. (**d** and **g**) Posterior cingulate: AD (*N*=9) and control (*N*=13). (**e** and **h**) Superior frontal gyrus: AD (*N*=23) and control (*N*=11). (**f** and **i**) Medial temporal gyrus: AD (*N*=16) and control (*N*=12). (**j**) A scheme summarizing tentative disease-protective and disease-promoting events associated with reduced *RGS2* expression in AD brain tissues. Squares on the left summarize our observations; adjacent squares summarize tentative related consquences (see the 'Discussion' section).

The expression levels of *RGS2* in LCLs from AD patients for whom cognitive scores were available (*n*=23) were examined for correlations with these scores (see Supplementary Table 1). A significant correlation (*R*=−0.555; *P*=0.006) was observed between the MMSE (Mini Mental State Examination) scores of AD patients and the *RGS*2 expression levels in their LCLs (Figure 4a). Moreover, a significant correlation (*R*=0.560; *P*=0.006) was observed between *RGS*2 expression levels and ADAS (Alzheimer's Disease Assessment Scale) scores (Figure 4b).

### GEO data mining indicates reduced *RGS2* expression in AD brain and blood

Data mining was performed for GEO data sets GSE5281 (ref. 28) from six postmortem brain regions (87 AD and 74 controls) and GSE63060 (ref. 29) from whole blood (145 AD, 80 MCI and 104 controls)—two data sets identified as the largest AD cohorts deposited on the NCBI Gene Expression Omnibus (GEO; see the 'Materials and methods' section). Both *RGS2* and *DLGAP1* exhibited significantly lower expression in postmortem AD brain tissues compared with matched controls of data set GSE5281.^28^ Moreover, *RGS2* also exhibited lower expression in whole blood data set GSE63060 (ref. 29) for both AD and MCI patients compared with healthy controls (FD=−1.2 and −1.3; *P*=0.000072 and 0.0000012, respectively; Figure 4c), suggesting that its low blood expression may serve as a peripheral MCI and AD biomarker.

## Discussion

Research on risk genes for late-onset AD (LOAD), the most common cause of dementia in the elderly, has been largely focused on the role of the ApoE4 genotype, the most notable genetic variation contributing to AD risk, whereas relatively few other genetic clues for this disease have been established. Yet, only about half of LOAD patients are ApoE4 carriers,^37^ suggesting that further genomic or epigenomic variations contribute to this neurodegenerative disease. These may include DNA sequence variations, gene or ncRNA transcripts, or epigenomic modifications that affect the sensitivity of brain cells to Aβ and/or tau. Our present study was aimed at discovering transcriptomic correlates for Aβ sensitivity as first step toward identifying LOAD risk genes.

The failure of genome-wide association studies to find major LOAD risk alleles besides ApoE4 suggests that transcriptomic and proteomic studies should be performed as, unlike genome-wide association studies, transcriptomic and proteomic studies also capture effects of microdeletions and individual epigenomic variations. In the present study, we have applied a microarray-based genome-wide transcriptomic approach, starting with LCLs from unrelated individual female donors, for searching gene expression variations that may correlate with LOAD risk due to more pronounced individual Aβ toxicity, and thereby, presumably, to LOAD. Notably, it has recently been demonstrated that LCLs capture life-course environmental epigenomic signatures.^38^ This genome-wide phase was followed by gene validation in a cohort of 28 AD patient LCLs compared with 32 LCLS from non-demented controls. We report 27 transcripts with expression levels associated with Aβ sensitivity of control LCLs (Table 1). Four of these genes, *RGS2*, *DLGAP1*, *BCHE* and SNORD116-13 exhibited significantly lower expression in the AD LCL cohort compared with healthy controls, whereas *DNASE1L3* exhibited higher expression in the latter (Figures 3a–e). Among these genes, only the expression of *RGS2* correlated with patient MMSE and ADAS cognitive scores (Figures 4a and b). Both *RGS2* and *DLGAP1* exhibited, in addition, lower expression in published gene expression data set (GSE5281; ref. 28) of postmortem AD compared with control brain tissues, and *RGS2* exhibited lower expression also in a whole blood data set (GSE63060; ref. 29) of AD and MCI patients compared with control (Figures 4c–i). Below, we discuss the LOAD risk relevance of these genes.

### *RGS2* expression

Our genome-wide transcriptomic profiling detected 2.1-fold reduced *RGS2* expression in a group of healthy donors LCLs exhibiting high Aβ sensitivity (*P*=0.035; Table 1). Next, we observed a 3.3-fold reduced *RGS2* expression in AD LCLs compared with matched controls (*P*=0.0008; Figure 3a). To our knowledge, this is the first report on reduced *RGS2* expression in AD cells. RGS proteins, comprising a family with 20 members, have key roles in synaptic signaling and neuronal plasticity: these proteins function as negative regulators of G-protein-coupled receptors (GPCR) signaling, acting as GTPase activating proteins for Gα subunits, thereby accelerating the turnoff of GPCR signaling.^39^ RGS2 has widespread brain expression,^40^ and its altered expression has been implicated in several neurodegenerative and psychiatric diseases.^41, 42, 43, 44, 45, 46, 47^ Unlike other RGS family members, *RGS2* is an immediate early gene, rapidly upregulated in response to stimuli evoking brain plasticity^39^ such as high frequency stimulation.^48^ Notably, *RGS2* expression was induced in rat hippocampus 2 h following acute electroconvulsive shock,^47^ and in human astrocytoma cells following heat shock or oxidative stress.^49^

*RGS2* was identified as key regulator of *LRRK2* (leucine-rich repeat kinase 2; also known as PARK8). *LRRK2* mutations cause shortening of the dendritic tree and are among the primary genetic causes of Parkinson's disease.^41, 45^ Reduced *RGS2* expression was observed in the striata of *LRRK2*-mutated and sporadic Parkinson's disease patients.^45^ In addition, *RGS2* rs4606 polymorphism is a risk allele for schizophrenia^43^ and is associated with antipsychotic-induced parkinsonism.^42^ RGS2 has also been suggested to be implicated in antioxidant defense.^50^

Decreased striatal *RGS2* expression has been suggested to be neuroprotective in Huntington's disease (HD).^44^ A similar compensatory response may underlie the lower *RGS2* expression observed in our AD LCLs (Figure 3a) and in postmortem AD brain tissues (Figures 4d–f). Notably, the decreased blood *RGS2* expression is already apparent at the MCI stage (Figure 4c) and prevails in AD blood samples. This may partly explain our observations that lower *RGS2* expression levels were correlated with better MMSE and ADAS scores (Figures 4a and b). Whatever the explanation, our data suggest that *RGS2* expression levels are implicated in AD pathology, either as causative or as disease-triggered protective mechanism, as has been suggested for its reduced expression in HD brains.^44^

Genes coding for GPCRs comprise the largest family in the human genome, with 791 different genes (~4% of the human exome), half coding for olfactory receptors.^51^ The activity of the olfactory receptors is tightly regulated by RGS family proteins, including RGS2.^52, 53^ Reduced olfactory sensing is a common feature in AD, observed already in some MCI patients.^54, 55, 56^ It is accompanied by reduced neuronal stem cell renewal in the olfactory epithelium, a tissue of central origin,^57^ owing to impaired neuronal stem cell migration and proliferation, possibly secondary to amyloid-β accumulation.^58^ Thus, it is plausible that reduced *RGS2* expression in MCI and AD patients represents a compensatory mechanism aimed at improving a deteriorating olfactory capacity.

Dysregulation of acetylcholine receptors, in particular the M1 muscarinic receptor, has received considerable interest in AD research, as this GPCR is implicated in memory consolidation^59, 60, 61^ and as acetylcholinesterase (AChE) inhibitors remain among first-line AD therapeutics. Decreased levels M1 muscarinic receptors have been demonstrated in several AD postmortem brain regions including CA1, temporal cortex and occipital cortex.^62, 63, 64^ Yet, M1 muscarinic signaling capacity was shown to be preserved in AD brain tissues.^65^ Notably, RGS2 has been shown to bind directly and selectively to the M1 muscarinic acetylcholine receptor (via the receptor's third intracellular loop) and modulate Gq/11alpha signaling^66^ resulting in suppression of M1 muscarinic receptor-mediated activation of KCNQ channels that in turn regulate neuronal excitability.^67^ It is therefore plausible that preserved M1 G-protein coupling capacity persists in AD brain tissues in spite of compromised acetylcholine levels in part owing to reduced *RGS2* expression that allows enhanced M1 muscarinic receptor signaling. This tentative scenario agrees with the above suggestion for a compensatory neuroprotective role of reduced brain *RGS2* expression, as also proposed for HD.^44^

Melatonin treatment has been suggested to ameliorate AD pathology and cognitive decline in animal models.^68, 69, 70^ Notably, melatonin production in the rat pineal gland was reduced following *Rgs2* transfection.^71^ Lower *RGS2* expression in AD LCLs, blood and brain (Figure 3a, Figures 4c–f) may indicate enhanced melatonin production. Moreover, melatonin treatment of multiple sclerosis patients upregulated *SIRT1* expression in their blood cells^72^ and reduced sepsis-induced brain injury through upregulation of *Sirt1* and *Bcl-2* in mice.^73^ Thus, lower *RGS2* expression in AD may allow higher pineal melatonin production and in turn improve neuroprotection.

Last, RGS2 has been reported as the only RGS family member that inhibits the mRNA translation into protein of eIF2Bε (eukaryotic initiation factor 2B ε subunit),^74^ a protein crucial for correct protein folding, a process dysfunctional in neurodegenerative disorders including HD, AD and prion diseases, and mutations in which cause childhood ataxia.^75^ This novel role of RGS2 supports its postulated defensive mechanism in both HD and AD, whereby reduced *RGS2* expression reflects an attempt to protect cells from misfolded protein accumulation by enhancing eIF2Bε translation.^76^

GPCRs are the largest gene family in the human genome (~800 members) and ~40% of current therapeutics are GPCR ligands.^77^ Our findings of the GPCR regulator *RGS2* as deregulated in AD LCLs (Figure 3a), and that its expression was correlated with AD patients' MMSE and ADAS scores (Figures 4a and b), are intriguing. Moreover, *RGS2* was found to be downregulated in published GEO data sets from postmortem AD brain tissues (Figures 4d–f), as well as in both AD and MCI peripheral blood (Figure 4c). A scheme summarizing tentative disease-protective and disease-promoting events associated with reduced *RGS2* expression is shown in Figure 4j. These observations attest to the complexity of the disease, with fundamental pathways led astray. It further highlights the need for innovative approaches to AD therapeutics.

### *DLGAP1* expression

*DLGAP1* expression was 2.1-fold lower in a group of healthy donors LCLs exhibiting high Aβ sensitivity (*P*=0.044; Table 1). We subsequently observed 2.8-fold reduced *DLGAP1* expression in AD LCLs compared with matched controls (*P*=0.042; Figure 3b). The proteins encoded by *DLGAP1* (also known as GKAP) along with *DLC2* take part in neuronal *N*-methyl-d-aspartate (NMDA)-receptor-associated scaffolding complex. NMDA glutamate receptors are strongly implicated in neurodegenerative diseases,^78^ and comprise the drug target of memantine, the first non-cholinesterase inhibitor FDA-approved AD drug.^79^ Interference of the *DLGAP1*–*DLC2* interaction inhibits NMDA receptor activity in dendritic spines.^80^ In turn, synaptic activity-induced *DLGAP1*–*DLC2* interaction in dendritic spines stabilizes the scaffolding complex and enhances the NMDA currents.^81, 82^

Of note, the NMDA receptor GluN1 subunit was increased 6-fold in postmortem AD frontal cortex compared with controls,^83^ supporting a key role for elevated NMDA receptor activity in glutamate-mediated neurodegeneration.^78^ Moreover, Aβ was shown to induce degradation of GKAP, the protein encoded by *DLGAP1.*^84^ Further studies are needed for clarifying how the latter observation is related to the reduced *DLGAP1* expression observed in our AD LCLs (Figure 3b).

The reduced *DLGAP1* expression in AD LCLs may represent, similarly to our above suggestions for *RGS2*, a compensatory mechanism for protecting against NMDA-mediated neuronal cell death. This tentative explanation needs further exploration, as the function of NMDA receptors in immune cells, although apparent, remains little studied.^85^

### *BCHE* expression

The expression of *BCHE*, coding for BChE, was 1.82-fold lower in the group of high Aβ sensitivity LCLs (Table 1). We subsequently observed 6.1-fold lower *BCHE* expression in AD LCLs compared with healthy controls (*P*=0.04; Figure 3c). BChE, along with AChE, comprise the targets of the first-generation AD drug rivastigmine and the (discontinued) first AD drug Tacrine. BChE was shown to prevent Aβ fibril formation,^86^ an observation that may explain the increased AD risk in carriers of BChE K, a variant with reduced enzymatic activity^87^ and found by a recent meta-analysis to pose increased AD risk.^88^ In support of our observations, significantly lower plasma BChE activity levels were reported in AD plasma samples compared with controls, and were associated with faster disease progression.^89^ Thus, our findings on reduced *BCHE* expression in control LCLs showing higher Aβ sensitivity (Figure 2c), as well as in AD LCLs (Figure 3c) seem to fit a putative protective role of BChE against Aβ toxicity, while questioning the benefit of mixed AChE/BChE inhibitors (such as rivastigmine) as AD therapeutics. Perhaps the benefit from inhibiting acetylcholine hydrolysis by AChE outweighs the disadvantage of BChE inhibition by such drugs. The impact of reduced *BCHE* expression on AD risk and pathology as well as potential clinical implications for choosing selective AChE inhibitors vs mixed AChE/BChE inhibitors in AD treatment should be further explored.

### SNORD116 transcripts

Two SNORD116 transcripts, SNORD116-13 and SNORD116-18, exhibited higher expression in the LCL group having higher Aβ sensitivity in the genome-wide expression profiling microarrays. The SNORD116-13 microarray data were validated by real-time PCR, albeit only with a trend for significance (*P*=0.07), indicating 1.70-fold higher expression levels in LCLs exhibiting high Aβ sensitivity. The same SNORD116-13 transcript showed 1.48-fold lower expression in AD LCLs vs healthy controls (*P*=0.0079; Figure 3d).

SNORD116 deletions cause Prader–Willi syndrome, a neurodevelopmental genetic disorder manifested in cognitive and behavioral deficits.^90^ SNORD transcripts are noncoding nucleolar RNAs acting similarly to transcription factors. SNORD116 was shown to be developmentally regulated in maturing neurons^91^ and its overexpression affects the expression of over 200 genes.^92^ SNORD116 transfection increased the expression of *MAP2* (microtubule-associated protein 2, an axonal marker) and *TUBB4* (tubulin beta-4 A chain), both important for microtubule assembly. The expression of both *MAP2* and *TUBB4* were decreased in postmortem posterior hypothalamus from Prader–Willi syndrome.^92^ Our findings on decreased SNORD116-13 expression in AD LCLs compared with controls, and increased expression in LCLs exhibiting higher Aβ sensitivity, suggest that some genes regulated by SNORD116 may be implicated in neurodegeneration, possibly by modifying cellular responses to chronic Aβ exposure.

### Strengths and constraints

Our observations suggest that the protein products of the genes discussed above may be implicated in the pathophysiology of sporadic AD. The correlations we have observed between their lower expression levels and higher Aβ sensitivity in healthy female donors LCLs suggest that their low expression may be among the causes rather than consequences for sporadic AD. Yet, considering reports of a compensatory neuroprotective role for reduced *RGS2* levels in HD, it may well be that the reduced *RGS2* expression levels that we observed in AD LCLs and postmortem brain reflect a similar compensatory mechanism in AD.

A key limitation of our study is that transcriptomic profiling assays were conducted in blood-derived cells, namely LCLs, rather than in neurons. Nonetheless, neuroimmune interactions have a key role in neurodegenerative diseases including AD,^93, 94, 95^ and the recent demonstration of a functional meningeal lymphatic system that drains cerebrospinal fluid to deep cervical lymph nodes^96^ emphasizes the relevance of neuroimmune interactions in neurodegenerative diseases. In favor of applying LCLs transcriptomic profiling for AD research are our observations on reduced *SIRT1* and *SARM1* expression in AD personal LCLs (Figures 3f–g), moreover, *SIRT1* expression was reduced in AD brains.^97^

Our hypothesis-free findings on lower expression of *RGS2* and *DLGAP1* in AD LCLs are supported by analysis of published gene expression data sets of postmortem AD brain tissues. *RGS2* expression levels were also lower in AD and MCI patients' blood (Figures 4c–i). Personal LCLs may thus serve, in the absence of neuronal tissues, as surrogate for brain cells, and may point to altered transcriptomic profiles that could be implicated in AD pathology.

## Conclusions

Our findings, based on a genome-wide transcriptomic search for genes implicated in Aβ sensitivity, show lower expression levels of several key regulatory genes. In particular, lower expression levels of *RGS2, DLGAP1* and *BCHE* are implicated in the higher Aβ sensitivity of LCLs from some individuals. Furthermore, lower expression levels of *RGS2* and *DLGAP1* were also found in LCLs of AD patients compared with non-demented control donors, as well as in two published gene expression data sets (GSE5281 and GSE63060) of postmortem AD brain tissues and in MCI and AD patients' blood. Taken together, we suggest the involvement of lower expression of *RGS2* and *DLGAP1* in AD pathophysiology. In particular, the potential diagnostic value of blood *RGS2* expression levels should be explored, as this reduction is already noticeable in blood samples of MCI patients. Further studies are required for elaborating the roles of these genes and their protein products, till now not implicated in AD, in the disease pathophysiology, as well as the potential of their expression levels as early AD biomarkers, and tentative utility as AD drug targets.

## Figures and Tables

**Table 1:** Genome-wide transcriptomic profiling comparing individual LCLs with high vs low Aβ_1__–42_ sensitivities (eight LCLs in each group; Affymetrix GeneChip Human Gene 2.1 ST arrays)

*Gene/transcript*	*Full name*	*Fold difference (high vs low)*	P*-value*
***DNASE1L3***	Deoxyribonuclease I-like 3	−2.40	0.003
*ABHD6*	Abhydrolase domain containing 6	−1.62	0.006
*MERTK*	c-mer proto-oncogene tyrosine kinase	−1.64	0.008
*PEX5L*	Peroxisomal biogenesis factor 5-like	−1.54	0.010
***FARP1***	FERM, RhoGEF (ARHGEF) and pleckstrin domain protein 1	1.69	0.010
LOC728419	Ubiquitin carboxyl-terminal hydrolase 17-like	−1.63	0.012
*OR5K4*	Olfactory receptor, family 5, subfamily K, member 4	−1.88	0.017
*FAH*	Fumarylacetoacetate hydrolase	1.51	0.021
*ZNF804A*	Zinc finger protein 804A	−1.77	0.023
*TNFRSF9*	Tumor necrosis factor receptor superfamily, member 9	−1.81	0.027
*RNU6-55*	RNA, U6 small nuclear 55	−1.64	0.029
*OR5H14*	Olfactory receptor, family 5, subfamily H, member 14	−2.40	0.031
***RGS2***	Regulator of G-protein signaling 2, 24 kDa	−2.14	0.035
*KDM5B*	Lysine (K)-specific demethylase 5B	−2.10	0.035
***SNORD116-13***	Small nucleolar RNA, C/D box 116-13	1.57	0.035
*CCL28*	Chemokine (C-C motif) ligand 28	−1.51	0.036
***INPP4B***	Inositol polyphosphate-4-phosphatase, type II, 105 kDa	−2.28	0.038
*PTPN14*	Protein tyrosine phosphatase, non-receptor type 14	−2.17	0.038
*TRNAU2*	Transfer RNA selenocysteine 2	1.64	0.038
*SNORD45C*	Small nucleolar RNA, C/D box 45C	1.58	0.041
*PHYHIPL*	Phytanoyl-CoA 2-hydroxylase interacting protein-like	−1.61	0.043
***DLGAP1***	Disks, large (Drosophila) homolog-associated protein 1	−2.10	0.044
*ANKRD20A11P*	Ankyrin repeat domain 20 family, member A11, pseudogene	−1.68	0.044
***PAG1***	Phosphoprotein associated with glycosphingolipid microdomain	−1.73	0.045
*SNORD116-18*	Small nucleolar RNA, C/D box 116-18	1.75	0.045
*GLIPR2*	GLI pathogenesis-related 2	−1.53	0.046
***BCHE***	Butyrylcholinesterase	−1.82	0.049

Abbreviations: Aβ, amyloid-β LCL, lymphoblastoid cells line.

The 27 listed transcripts differed by >1.5-fold with *P*<0.05 in eight LCLs exhibiting high Aβ_1__–42_ sensitivity compared with eight LCLs exhibiting low Aβ_1__–42_ sensitivity (as shown in Figure 1c). Genes are arranged by increasing *P*-values. The expression differences for eight selected genes (in bold font) were tested in the same RNA samples by real-time PCR experiments (Figures 2a–f) and further tested in Alzheimer's disease LCLs (Figures 3a–e; Supplementary Table 2).
