# *RGS2* expression predicts amyloid-β sensitivity, MCI and Alzheimer's disease: genome-wide transcriptomic profiling and bioinformatics data mining

**DOI:** 10.1038/tp.2016.228

**Published:** 2017-02-14

**Authors:** A Hadar, E Milanesi, A Squassina, P Niola, C Chillotti, M Pasmanik-Chor, O Yaron, P Martásek, M Rehavi, D Weissglas-Volkov, N Shomron, I Gozes, D Gurwitz

**Correction to:** *Translational Psychiatry* (2016) **6**, e909; doi:10.1038/tp.2016.179; published online 4 October 2016

In the online version of this paper, the second chart that appears in the Materials and Methods section, 'Real-time PCR' subsection, is incorrect. The correct chart appears below:


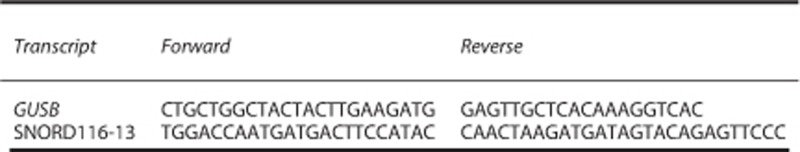


The publisher regrets this error.

