# Supplementary material for: RGS2 expression predicts amyloid-β sensitivity, MCI and Alzheimer's disease: genome-wide transcriptomic profiling and bioinformatics data mining
Source: Transl Psychiatry. 2016 Oct 4;6(10):e909–. doi: 10.1038/tp.2016.179 (PMC5315547; doi:10.1038/tp.2016.179)
Supplement: Supplementary Information [file tp2016179x1.doc]

**Supplementary Information**

***RGS2* expression predicts amyloid-β sensitivity, MCI and Alzheimer’s disease: genome-wide transcriptomic profiling and bioinformatics data mining**

Adva Hadar, MSc^1^, Elena Milanesi, PhD^1,2^, Alessio Squassina, PhD^3^, Paola Niola, MD^3^, Caterina Chillotti, MD^4^, Metsada Pasmanik-Chor, PhD^5^, Orly Yaron, PhD^6^, Pavel Martásek, MD, DSc^7^, Moshe Rehavi, PhD^8^, Daphna Weissglas-Volkov, PhD^9^, Noam Shomron, PhD^9,10^, Illana Gozes, PhD^1,10^, and David Gurwitz, PhD^1,10^

^1^Department of Human Molecular Genetics and Biochemistry, Sackler Faculty of Medicine, Tel-Aviv University, Tel-Aviv, Israel

^2^Genetic Unit, IRCCS Istituto Centro San Giovanni di Dio Fatebenefratelli, Brescia, Italy.

^3^Department of Biomedical Sciences, University of Cagliari, Cagliari, Italy

^4^Unit of Clinical Pharmacology of the University Hospital of Cagliari, Italy

^5^Bioinformatics Unit, George Wise Faculty of Life Sciences, Tel-Aviv University, Tel-Aviv, Israel

^6^The Genomic Analysis Laboratory, Sackler Faculty of Medicine, Tel-Aviv University, Tel-Aviv, Israel

^7^Department of Pediatrics and Adolescent Medicine, First Faculty of Medicine, Charles University in Prague and General University Hospital in Prague, Czech Republic.

^8^Department of Physiology and Pharmacology, Sackler Faculty of Medicine, Tel-Aviv University, Ramat Aviv, 69978, Tel-Aviv, Israel

^9^Department of Cell and Developmental Biology, Sackler Faculty of Medicine, Tel-Aviv University, Tel-Aviv, Israel

^10^Adams Super Center for Brain Studies, and Sagol School of Neuroscience, Tel-Aviv University, Tel-Aviv, Israel

**Supplementary Table 1: (a)** Alzheimer’s disease patient demographic data. Sex, age, age at first diagnosis, MMSE and ADAS measures are shown for 28 patients.

| ***ID sample*** | ***Sex*** | ***Age at sampling***  ***(years)*** | ***Age at onset***  ***(years)*** | ***MMSE*** | ***ADAS*** |
| --- | --- | --- | --- | --- | --- |
| 1202 | M | 91 | 88 | 20.4 | 13 |
| 1162 | F | 90 | 83 | 14.4 | 16.3 |
| 1088 | F | 89 | 86 | 15.4 | 25.9 |
| 1092 | F | 89 | 84 | 14.8 | 15.6 |
| 1170 | F | 88 | 84 | NA | NA |
| 1150 | F | 82 | 79 | 20.5 | 17.6 |
| 1215 | F | 82 | 80 | 14.5 | 27 |
| 1131 | M | 80 | 78 | 25.7 | 12.6 |
| 1098 | M | 79 | 74 | 23 | 12.2 |
| 1178 | M | 79 | 75 | 25.3 | 9.3 |
| 1132 | F | 77 | 75 | 15 | 29.6 |
| 1149 | F | 77 | 75 | 20.7 | 6.6 |
| 1130 | F | 76 | 75 | 20.7 | 10.6 |
| 1219 | F | 76 | 74 | 22 | 13.6 |
| 1110 | F | 75 | 73 | 24 | 11.9 |
| 1121 | F | 75 | 74 | 19.7 | 15.6 |
| 1135 | F | 75 | 70 | 22.3 | 17.2 |
| 1214 | F | 74 | 73 | 16.3 | 14.9 |
| 1090 | F | 73 | 71 | 21.4 | 14.9 |
| 1187 | F | 73 | 70 | 18.7 | 20 |
| 1229 | F | 73 | 71 | 20.4 | 20.3 |
| 1235 | F | 73 | 71 | 21.3 | 17 |
| 1173 | F | 71 | 69 | 19.3 | 23.3 |
| 1197 | F | 71 | 69 | 19.7 | 10.6 |
| 1100 | F | 70 | 66 | 14.3 | 23.2 |
| 1118 | F | 68 | 67 | 21.4 | 19.7 |
| 1141 | F | 75 | 73 | 22 | 23 |
| 1145 | F | 68 | 66 | NA | NA |

**(b)** Demographic data for 32 healthy adult controls. First 16 control LCLs were from Cagliari, Italy, while the rest were from the NLGIP biobank (see Methods).

| ***ID sample*** | ***Sex*** | ***Age***  ***(years)*** |
| --- | --- | --- |
| 1012 | F | 56 |
| 1016 | F | 47 |
| 1060 | F | 79 |
| 1376 | F | 56 |
| 1377 | M | 82 |
| 1378 | M | 70 |
| 1379 | F | 74 |
| 1380 | M | 74 |
| 1446 | F | 63 |
| 1466 | F | 34 |
| 1706 | F | 35 |
| 1728 | F | 48 |
| 1744 | F | 46 |
| 1791 | F | 47 |
| 1793 | F | 40 |
| 1799 | F | 34 |
| 1728 | F | 22 |
| 1516 | F | 75 |
| 1370 | F | 24 |
| 1515 | F | 88 |
| 1389 | F | 28 |
| 1978 | F | 76 |
| 1518 | F | 82 |
| 1130 | F | 72 |
| 1146 | F | 21 |
| 6037 | F | 77 |
| 1826 | F | 27 |
| 1823 | F | 25 |
| 1549 | F | 73 |
| 1754 | F | 25 |
| 1801 | F | 27 |
| 1128 | F | 31 |

**(c)** Demographic data for Alzheimer’s disease patients and controls. The data are shown as mean±SD.***P<0.001

| ***Group*** | ***N*** | ***Age*** | ***Sex*** | |
| --- | --- | --- | --- | --- |
|  |  |  | ***F*** | ***M*** |
| Alzheimer’s disease patients | 28 | 77.46±6.7*** | 24 | 4 |
| Healthy adult | 32 | 51.81±22.3 | 29 | 3 |

**Supplementary Table 2.** Expression levels of candidate genes in 28 AD and 32 healthy control LCLs, as determined by real-time PCR (see Methods). Genes are listed by increasing P values. See Methods for details.

| ***Gene*** | ***Fold- difference AD vs. control LCLs*** | ***P- value*** | ***Annotation*** |
| --- | --- | --- | --- |
| ***RGS2*** | -3.3 | 0.0008 | a modulator of LRRK2 activity and neuronal toxicity |
| ***DNASE1L3*** | 1.9 | 0.013 | Activated in apoptosis |
| ***BCHE*** | -6.1 | 0.040 | Butyrylcholinesterase attenuates amyloid fibril formation in vitro |
| ***DLGAP1*** | -2.8 | 0.042 | N-methyl-D-aspartate (NMDA)-receptor-associated scaffolding complex |
| ***FARP1*** | -1.8 | 0.048 | Encoded protein functions in neurons to promote dendritic growth |
| ***PAG1*** | -1.1 | 0.112 | Associated with pathogenesis of Alzheimer's disease |
| ***INPP4B*** | -1.46 | 0.174 | Expression controlled by estrogen |


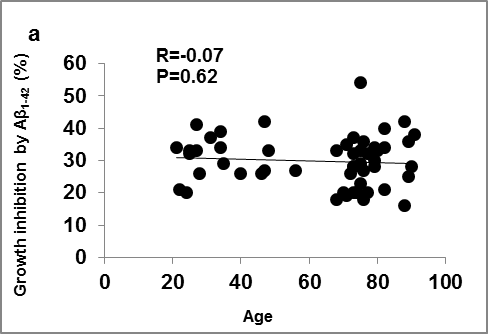


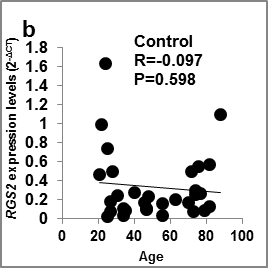

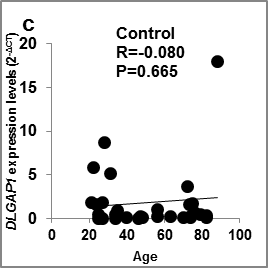

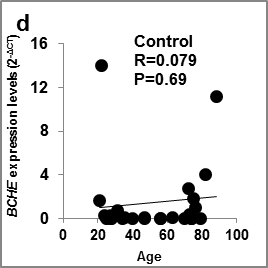

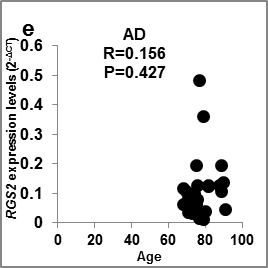

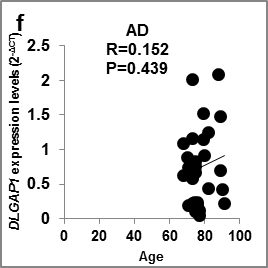

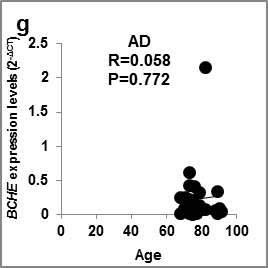


**Supplementary Figure 1.** Lack of correlations between donor age and: **(a)** Growth inhibition by 8 μM Aβ_1-42_ in individual LCLs (n=53) **(b,e)** *RGS2* expression in Control LCLs (n=32), AD LCLs (N=28). **(c,f)** *DLGAP1* expression in Control LCLs (n=32), AD LCLs (N=28). **(d,g)** *BCHE* expression in Control LCLs (n=28), AD LCLs (N=27).


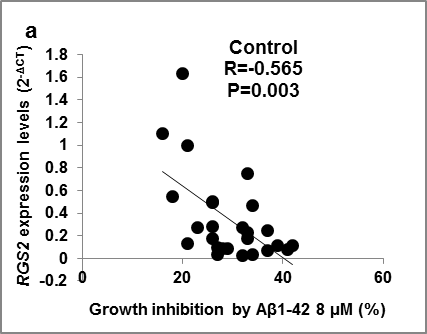

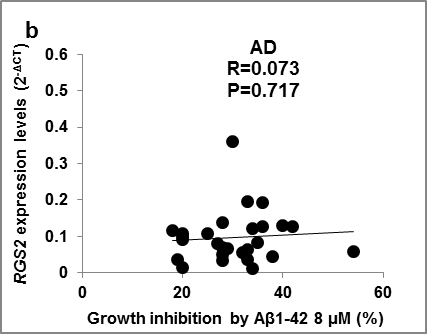


**Supplementary Figure 2.** Correlations between the expression levels of *RGS2* and growth inhibition by Aβ_1-42_ in: **(a)** individual healthy control LCLs (n=26). **(b)** Alzheimer’s disease LCLs (n=27).


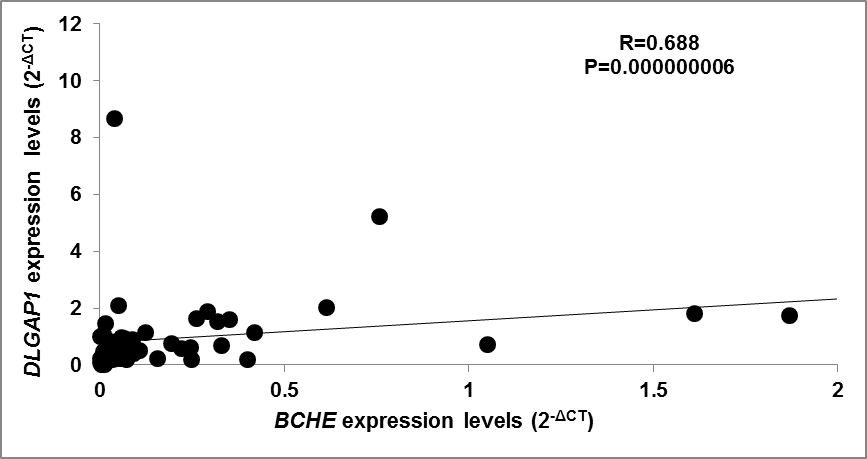


**Supplementary Figure 3.** Correlations between the expression levels of *DLGAP1* and *BCHE* in individual LCLs (n=55). LCLs were pooled for AD and controls.


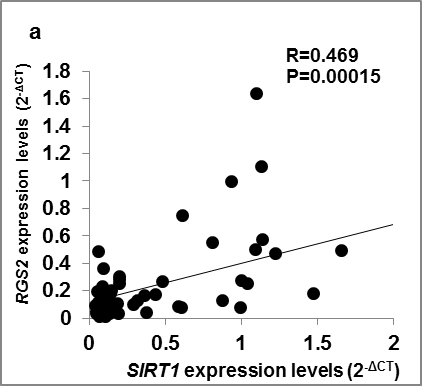

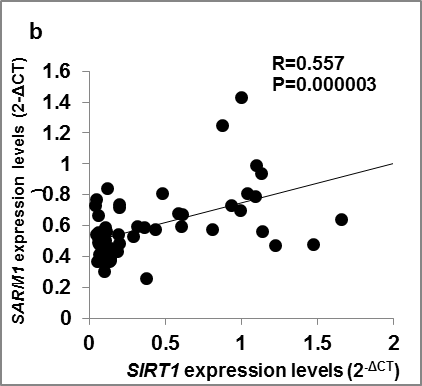

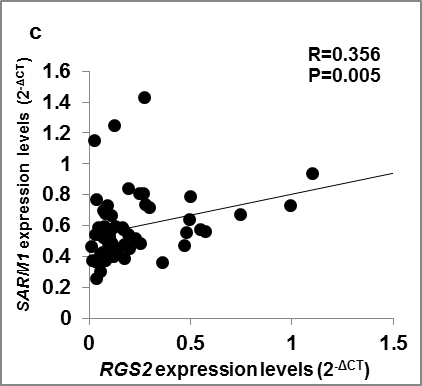

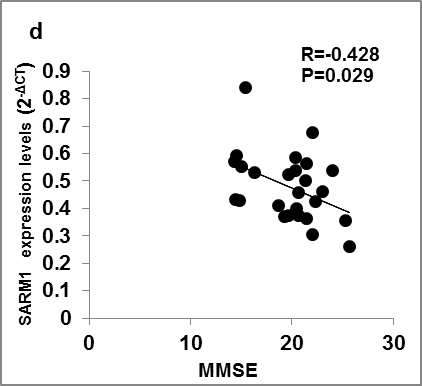


**Supplementary Figure 4.** Correlations between the expression levels of *SIRT1* in individual LCLs (n=60; pooled AD and controls) and: **(a)** *RGS2*. **(b)** *SARM1*. **(c)** Correlation between the expression levels of *SARM1* and *RGS2* in individual LCLs (n=60; pooled AD and controls). **(d)** Correlation between the LCL expression levels of *SARM1* and MMSE scores in Alzheimer’s disease patients (n=26).
